# Structure of the cytoplasmic domain of SctV (SsaV) from the *Salmonella* SPI-2 injectisome and implications for a pH sensing mechanism

**DOI:** 10.1016/j.jsb.2021.107729

**Published:** 2021-06

**Authors:** Teige R.S. Matthews-Palmer, Nayim Gonzalez-Rodriguez, Thomas Calcraft, Signe Lagercrantz, Tobias Zachs, Xiu-Jun Yu, Grzegorz J. Grabe, David W. Holden, Andrea Nans, Peter B. Rosenthal, Sarah L. Rouse, Morgan Beeby

**Affiliations:** a Department of Life Sciences, Imperial College London, London SW7 2AZ, United Kingdom; b Structural Biology of Cells and Viruses Laboratory, The Francis Crick Institute, London, United Kingdom; c MRC Centre for Molecular Bacteriology and Infection, Imperial College London, London, United Kingdom

**Keywords:** SctV, Specificity switch, cryo-EM, Injectisome, Type III secretion system, Molecular dynamics

## Abstract

Bacterial type III secretion systems assemble the axial structures of both injectisomes and flagella. Injectisome type III secretion systems subsequently secrete effector proteins through their hollow needle into a host, requiring co-ordination. In the *Salmonella enterica* serovar Typhimurium SPI-2 injectisome, this switch is triggered by sensing the neutral pH of the host cytoplasm. Central to specificity switching is a nonameric SctV protein with an N-terminal transmembrane domain and a toroidal C-terminal cytoplasmic domain. A ‘gatekeeper’ complex interacts with the SctV cytoplasmic domain in a pH dependent manner, facilitating translocon secretion while repressing effector secretion through a poorly understood mechanism. To better understand the role of SctV in SPI-2 translocon-effector specificity switching, we purified full-length SctV and determined its toroidal cytoplasmic region’s structure using cryo-EM. Structural comparisons and molecular dynamics simulations revealed that the cytoplasmic torus is stabilized by its core subdomain 3, about which subdomains 2 and 4 hinge, varying the flexible outside cleft implicated in gatekeeper and substrate binding. In light of patterns of surface conservation, deprotonation, and structural motion, the location of previously identified critical residues suggest that gatekeeper binds a cleft buried between neighboring subdomain 4s. Simulations suggest that a local pH change from 5 to 7.2 stabilizes the subdomain 3 hinge and narrows the central aperture of the nonameric torus. Our results are consistent with a model of local pH sensing at SctV, where pH-dependent dynamics of SctV cytoplasmic domain affect binding of gatekeeper complex.

## Highlights

• CryoEM of a full-length type III secretion system SctV resolves cytoplasmic but not transmembrane domains.
• MD simulations show SctV protomers flexibly hinge.
• Acidification expands the SctV ring by altering interprotomer interactions.

## Graphical abstract

**Figure:**
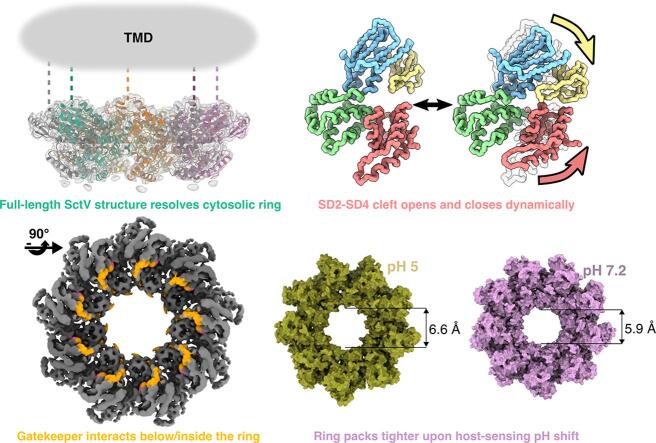

## 1 Introduction

**Fig. 1:**
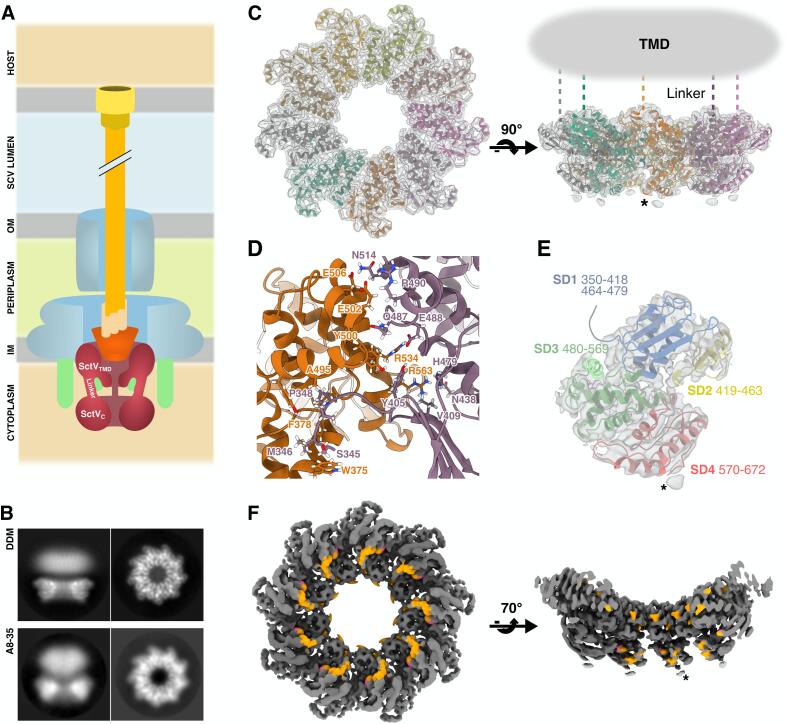
SctV_C9_ cryo-EM structure. A. Schematic representation of the T3SS injectisome. B. 2D classification averages showing side and top views of DDM-solubilised SctV (top row) and A8-35-solubilised SctV (bottom row). C. SctV_C_ model fitted into our Coulomb potential map, top (left) and side (right) views. SctV_N_ and linker are depicted by cartoons. D. Cartoon and stick representation of the interactions in the interface between SctV_C_ protomers. The 5 last residues of the linker domain (345–349) participate in the interaction by lying on top of the neighbouring protomer. E. Side view of a SctV_C_ monomer depicting arrangement of its subdomains: SD1, blue; SD2, yellow; SD3, green; SD4, red. This colour scheme is retained in subsequent figures. Note the density blob on the cytosolic face of the ring, corresponding to the presumed flexible nine-residue C-terminal tail of SctV. F. Coulomb potential map of SctV_C_. Residues involved in gatekeeper complex binding (Yu et al., 2018) are highlighted in orange, V632 is coloured magenta. Asterisks in panels B, D and E indicate the predicted region of the C terminal tail.

Bacterial type III secretion systems (T3SSs) are required for virulence by many bacteria. T3SSs are multiprotein inner membrane complexes that export virulence proteins and self-assembling components of one of two types of trans-periplasmic molecular machines through hollow axial structures. The better-characterized of the T3SS-based molecular machines, flagella, are used for motility and other aspects of pathogenesis (Chaban et al., 2015). Flagellar T3SSs first export self-assembling components of the rod (a trans-periplasmic driveshaft) and a short extracellular “hook” (a universal joint that redirects flagellar torque). Upon the hook reaching a predetermined length, the T3SS switches export specificity to export subunits of the multi-micron long flagellum, which forms a helical propellor for motility. The second type of T3SS-based molecular machines, injectisomes (sometimes referred to simply as “type III secretion systems”, or non-flagellar T3SSs), are used by a wide variety of bacterial pathogens of animals and plants but differ functionally to flagella as virulence protein delivery machines that evoke molecular hypodermic syringes (Fig. 1A). The injectisome T3SS first exports proteins that form a rigid hollow needle. Upon the needle reaching a predetermined length, the T3SS switches specificity to export translocon proteins that cap the needle and form a pore in host cell membranes (Journet et al., 2003). Upon host cell contact, the T3SS switches specificity again to export virulence proteins (effectors) through the translocon pore into the host cell where they hijack host cell physiology to benefit the pathogen (Figueira and Holden, 2012).

The control of which proteins are secreted, and when, is key to effective pathogenesis, and better understanding of these mechanisms is foundational to understanding T3SS-based diseases (McShan and De Guzman, 2015). The first secretion switch is well-studied in flagellar and injectisome T3SSs: a molecular ruler (SctP) is sporadically secreted; if the length of the hook or needle exceeds that of the ruler, a conformational change in one of the T3SS proteins switches export specificity (Journet et al., 2003). The second secretion switch remains relatively poorly understood. This switch is characterized only in injectisome T3SSs, where detection of host cell contact triggers translocation of effector proteins. The system in which this second switch is best characterized is the *Salmonella* Pathogenicity Island 2 (SPI-2) injectisome from *Salmonella enterica* Serovar Typhimurium, which enables delivery of effectors across intracellular *Salmonella-*containing vacuoles (SCVs) to enable *Salmonella* replication. Initial expression and assembly of SPI-2 injectisome components is triggered by acidification and nutrient deprivation of the SCV after bacterial invasion or phagocytosis (Rappl et al., 2003, Chakravortty et al., 2005). The second switch is triggered by sensing the neutral pH of host cell cytoplasm through the translocon pore (Yu et al., 2010). Nevertheless, the mechanism by which this pH shift is sensed remains unclear.

The protein at the heart of both T3SS secretion and secretion specificity belongs to the SctV/FlhA family (injectisome and flagellar system names respectively). While only injectisome members have been given unified Sct names (Hueck, 1998), the SctV/FlhA family is conserved across all T3SSs of both flagella and injectisomes (Abby and Rocha, 2012), which assembles early into the inner membrane (Diepold and Wagner, 2014), and is central to T3SS assembly and function (Erhardt et al., 2017). In the SPI-2 T3SS, SctV is often referred to by its original species-specific name SsaV; here we call it SctV^SPI-2^. The ~75 kDa SctV is composed of an N-terminal transmembrane domain functioning as a proton-protein antiporter export gate, and a C-terminal cytoplasmic domain (SctV_C_) implicated in funneling selected substrates to the export gate.

SctV_C_’s substrate specificity is mediated by a trimeric “gatekeeper” complex (Portaliou et al., 2017, Yu et al., 2018) formed of SctW (in SPI-2, SsaL) and a heterodimeric chaperone (in SPI-2, SsaM and SpiC (Yu et al., 2004), lacking unified Sct nomenclature). The gatekeeper binds both SctV_C_ and mid-stage substrate chaperones, suggesting it adapts SctV_C_ for mid-stage substrates; gatekeeper dissociation or disruption permits effector secretion (Portaliou et al., 2017, Yu et al., 2018). Upon detection of the neutral pH of the host cell, the gatekeeper dissociates (Yu et al., 2010). SctV^SPI-2^ also features a short C-terminal extension not found in other branches of the SctV protein family, apparently required for efficient control of substrate specificity by the gatekeeper (Yu et al., 2018).

How the gatekeeper works is unknown, with three key gaps in our understanding of the gatekeeper-mediated second specificity switch by SctV. How does SctV detect changes in pH? Through direct deprotonation of SctV, communicated by other proteins of the injectisome, or through a more indirect process? What is the role of the unique SPI-2 C-terminal extension? What conformation or dynamic changes in SctV arise, and how do they cause changes in export specificity? Here we describe our work to determine the structure of SctV^SPI-2^ and simulate its dynamics at the different pH values that determine the substrate switch. Our findings are most consistent with pH directly modulating the dynamics of SctV_C_ to affect gatekeeper affinity.

## 2 Materials and methods

### 2.1 Data availability

The pQlinkN-SctV-6His plasmid generated in this study is available upon request.

The atomic coordinates of our SctV_C_ model and SctV_9_ Coulomb potential map have been deposited in the Protein Data Bank (PDB) (accession number 7AWA) and the Electron Microscopy Data Bank (EMDB) (accession number 11928), respectively.

### 2.2 Bacterial strains

SctV^SPI-2^ was expressed in *Escherichia coli* C41 harboring the pQlinkN-SctV-6His, grown in LB broth at 37 °C containing 100 g/ml ampicillin.

### 2.3 Cloning

Primers SctV_f_ (CGGGATCCCGTTCATGGTTAGGTGAGGGAG) and SctV_r_ (TCAAGCTTAGTGGTGGTGGTGGTGGTGTTCTTCATTGTCCGCCAACTCC) were used to amplify *ssaV-6His* from *S.* Typhimurium strain 12,023 genomic DNA. The PCR product was digested with BamHI and HindIII and ligated into the same sites of pQLinkN (Scheich et al., 2007) to create pQlinkN-SctV-6His. The construct was verified by DNA sequencing.

### 2.4 SctV expression and purification

The pQlinkN-SctV-6His plasmid was transformed into an *Escherichia coli* C41 expression strain. C41 cells, taken from an LB-Amp agar plate, were grown in lysogeny broth with 100 μg/ml ampicillin (LB-Amp) shaking at 37 °C overnight for starter cultures. Batches of 6L or 9L of expression culture were grown. 50 ml of turbid starter culture was diluted into 1 L of fresh LB-Amp in each baffled 2 L flask and grown shaking at 220 rpm and 37 °C to an OD_600_ = 0.6. IPTG was then added to a final concentration of 1 mM and cultures were incubated at 18 °C, shaking at 220 rpm for 24 h. Cells were harvested by centrifugation at 4500*g* for 30 min.

Cell pellets were resuspended in 150 ml lysis buffer (50 mM Tris-HCl pH 8.0, 150 mM NaCl, 1 tablet of EDTA-free protease inhibitor cocktail), homogenised manually with a PTFE-glass homogeniser and kept on ice at all times. 2 μl of 2500 U/ml DNAse-I was added to the cell suspension prior to lysis in a Constant Systems cell disruptor cooled to 4 °C at 25 kpsi in two successive rounds. Cell debris was removed by centrifugation at 39,000*g* for 30 min at 4 °C. Supernatant was centrifuged at 195,000*g* for 1 h at 4 °C to separate membranes. Membrane pellets were homogenised using a teflon-glass manual homogeniser in resuspension buffer (50 mM Tris-HCl pH 8.0, 150 mM NaCl, 10 mM imidazole, 5 mM DTT) supplemented with 10% glycerol, flash frozen by pipetting membrane suspension droplets directly into liquid nitrogen, and stored at −80 °C. Frozen membrane suspension was thawed in 50 ml of resuspension buffer supplemented with 1% DDM and incubated on a roller for 1 h at 4 °C. Insoluble components were removed by centrifugation at 195,000*g* for 30 min at 4 °C and filtering the supernatant through a 0.2 μm syringe filter of cellulose acetate.

Affinity purification was performed on 5 ml HisTrap FF columns. The column was equilibrated in wash buffer (50 mM Tris-HCl pH 8.0, 150 mM NaCl, 10 mM imidazole, 0.03%DDM, 5 mM DTT) and 50 ml solubilised protein solution loaded at approximately 5 ml/min. The HisTrap column was then connected to an ÄKTA Pure system at 5 ml/min, and washed with 100 ml of wash buffer. Elution buffer (50 mM Tris-HCl pH 8.0, 150 mM NaCl, 250 mM imidazole, 0.03% DDM, 5 mM DTT) was applied as a 20-ml-broad gradient against wash buffer and the eluate collected in 1 ml fractions. SDS-PAGE was used to identify and pool the fractions containing high concentration of SctV. Buffer was exchanged by diluting the pooled fractions 20x with SEC1 buffer (50 mM Tris-HCl pH 8.0, 50 mM NaCl, 0.03% DDM, 5 mM DTT) and then concentration in Amicon Ultra regenerated cellulose membrane 100 kDa centrifugal filters to 500 μl. Size-exclusion chromatography (SEC) was performed on a Superose 6 10/300 column equilibrated in SEC1 buffer at a 0.3 ml/min flowrate. The eluate was fractionated and inspected by SDS-PAGE and negative stain EM to assess the quality of SctV particles across the peak.

### 2.5 Amphipol exchange

DDM-solubilised sample purified by SEC was pooled, amphipol A8-35 was added at 1:2 mass ratio (amphipol:SctV), and incubated at 4 °C rocking for 4 h. Hydrated polystyrene biobeads were added at ~20:1 mass ratio (biobeads:DDM) to absorb DDM from the solution and incubated rocking overnight at 4 °C. The sample was removed from the biobeads by gentle centrifugation through a needle hole made in the bottom of a microfuge tube and concentrated to 500 μl in an Amicon Ultra regenerated cellulose membrane 100 kDa centrifugal filter. SEC was performed on the amphipol-exchanged sample in a Superose 6 10/300 column equilibrated with SEC2 buffer (50 mM Tris-HCl pH 8.0, 50 mM NaCl, 5 mM DTT) at a 0.3 ml/min flowrate. The eluate was fractionated and inspected by SDS-PAGE and negative stain EM.

### 2.6 Negative stain EM

Samples were diluted or concentrated to approximately 0.05 mg/ml. Continuous carbon EM grids were glow discharged in air for 30–40 s. 3 μl sample was applied onto grids and washed immediately with 40 μl of 2% uranyl acetate, then blotted with Whatman filter paper and dried in air. Images of negative stained SctV were collected in manual low dose mode at 120 keV on an FEI T12 with LaB_6_ source and TVIPs CCD camera, 42,000x magnification (pixel size of 3.2 Å), defocus range 1.5–2.5 μm.

### 2.7 Cryo-EM sample preparation

Quantifoil R2/2 holey grids with a 3-nm continuous carbon film overlaid were glow discharged in air for 30–40 s before applying 3 μl of the DDM-SctV sample at ~1 mg/ml, blotted and plunge frozen using a Vitrobot MkIV at 100% humidity with zero wait time. For A8-35-SctV, UltrAufoil R2/2 holey gold grids without any continuous support layer were used, and the method described in (Passmore and Russo, 2016), involving cleaning grids, equilibrating filter papers in the humidity chamber, and blotting at 4 °C with zero wait time was adopted with relative success.

### 2.8 Data collection and image processing

A8-35-SctV was imaged on an FEI F20 with Schottky FEG at 200 keV and FEI Falcon-2 direct detector. Exposure movies were collected using EPU for low-dose automation with 2.5–4 μm underfocus, 80 e^−^/Å^2^ fluence and a pixel size of 1.65 Å. Motion correction was performed with Motioncor2, defocus estimated with CTFFIND4. Xmipp3 interactive particle picker was used to curate 3000 particles which were classified in Relion 3.0 (Zivanov et al., 2018) producing templates for autopicking. 21,000 particles after classification and removal of bad picks were used to generate 2-D class averages in Relion 3.0.

High-resolution data of DDM-SctV on continuous carbon was collected on an FEI Krios equipped with Schottky FEG operating at 300 keV and a Gatan K2 direct detector with energy filter slit at 20 eV, 100 μm objective aperture, 1.5–4 μm underfocus, and 70 e^−^/Å^2^ fluence. Exposure movies were collected in counting mode, generating 40 frames over 13 s exposures, with pixel size of 1.08 Å. Motion correction was performed with Motioncor2 (Zheng et al., 2017), defocus estimation with CTFFIND4 (Rohou and Grigorieff, 2015). Xmipp3 interactive particle picker (de la Rosa-Trevín et al., 2013) was trained to pick close to 1 million particles from over 6000 micrographs, which was reduced to 361,000 particles by 2-D and 3-D classification in Relion 3.0. Particle polishing and refinement of the cytoplasmic domain within a soft mask was performed in Relion 3.0 and postprocessing estimated a B-factor of −151 Å^2^.

### 2.9 Model building

The reference sequence for SctV in this study is found under UniProt accession P74856. Using it as input for the Phyre2 server (Kelley et al., 2015) using default settings, a SctV_C_ initial model was generated. ISOLDE (Croll, 2018) was used to correct model geometry and fit the model to the Coulombic potential map. Several iterations of manual and automatic refinement using Coot (Emsley and Cowtan, 2004) and Phenix (Afonine et al., 2018) were performed to solve atomic clashes and geometric and density fitness errors. All-atom and model-to-map validation was performed using MolProbity (Chen et al., 2010).

### 2.10 Molecular dynamics simulations

All the steps were performed using GROMACS 2020.3 (Abraham et al., 2015). The protonation states of SctV_C9_ and SctV_C_ at pH 5.0 and 7.2 were predicted by ProteinPrepare (Martínez-Rosell et al., 2017). SctV_C9_ and SctVC were simulated in 20 × 20 × 16 and 12 × 12 × 12 nm boxes respectively, both using the CHARMM36 force field and TIP3P water models (Huang and MacKerell, 2013). Both boxes were fully solvated with water molecules and 150 mM NaCl, with a system net charge of 0. All the simulation steps were performed using the Particle-Mesh Ewald algorithm for electrostatic interactions with a cut-off of 1.2 nm. Both structures underwent energy minimization using 4000 steps using the steepest descent algorithm, with a step size of 0.1 Å, performing neighbour searching every 10 steps, and equilibrated for 1.5 ns with a time step of 1 fs, performing neighbour searching every 20 steps. Temperature coupling was performed using the Nose-Hoover algorithm. Pressure coupling was performed using the Parrinello-Rahman algorithm. The length of each simulation is summarised in Table S2.

### 2.11 Molecular dynamics simulations data analysis

Clustering analyses were performed using the gmx cluster tool on the whole trajectories using the gromos clustering algorithm and a 0.4 nm cutoff. The SD2-SD4 distance tracking was performed on all monomeric SctV_C_ simulations using gmx mindist and analysed using the Peptides (Osorio et al., 2015) package for R. The aperture of nonameric SctV_C_ was measured for all replicates using the gmx distance tool and analysed using the same R package. All of the gmx tools are included in GROMACS 2020.3 (Abraham et al., 2015). The PCA was performed using the pca tool in the R bio3d package (Grant et al., 2006) using the protein backbone and whole trajectories of the monomeric SctV_C_ simulations as input.

### 2.12 Visualisation of structural data, figures and other analyses

All panels involving atomic models or coulombic potential maps were built using ChimeraX (Goddard et al., 2018). Solvent accessible surfaces were calculated using GetArea (Fraczkiewicz and Braun, 1998) with a probe radius of 1.4 Å. The conservation analysis of SctV_C_ was performed using the Consurf webserver using the default parameters (Ashkenazy et al., 2016). Visual inspection of all the simulations trajectories and production of supplementary videos used VMD (Humphrey et al., 1996). Multiple sequence alignment (MSA) was performed using Clustal Omega (Sievers and Higgins, 2014) with default parameters and using as input the following SctV sequences as deposited in UniProt: SPI-2 (P74856), *E. coli* (Q7DB70), *Y. pestis* (A0A5P8YBD9)*, S. flexneri* (P0A1I5), SPI-1 (P0A1I3), *P. acanthamoebae* (F8KXZ3)*, B. pseudomallei* (Q3JL10) and *X. theicola* (A0A2S6ZDT7). The visual representation of the MSA was performed using the desktop version of pyBoxShade (available in https://github.com/mdbaron42/pyBoxshade). Secondary structure predictions were performed using PSIPRED 4.0 (Jones, 1999), JPred 4 (Drozdetskiy et al., 2015), PredictProtein (Yachdav et al., 2014) and RaptorX-Property (Wang et al., 2016) with default parameters in their respective webservers.

## 3 Results

### 3.1 Purification of nonameric rings of full-length SctV

Toward understanding how SctV orchestrates switching from translocon to effector secretion, we purified *Salmonella* SctV^SPI-2^ for structure determination. SctV is composed of an N-terminal transmembrane domain (TMD) SctV_N_ joined by a linker to a C-terminal cytosolic domain SctV_C_. We expressed full-length SctV fused to a C-terminal 6xHis tag in *Escherichia coli* C41. We extracted SctV from membranes using Dodecyl-beta-D-maltoside (DDM) and purified it using affinity and size exclusion chromatography (SEC) with DDM to stabilize the TMD. SctV eluted from the SEC column at the volume expected for a ~680 kDa complex, corresponding to a SctV nonamer (SctV_9_) (Fig. S1A). Negative stain EM imaging confirmed rings resembling the ~17 nm-diameter and ninefold symmetry of SctV observed *in situ* and in previously reported SctV cytosolic domain structures (Chen et al., 2011, Abrusci et al., 2013, Inoue et al., 2019, Butan et al., 2019, Majewski et al., 2020) (Fig. S1C).

### 3.2 Structure determination of SctV_C_

We used single particle analysis cryo-EM to determine the structure of SctV. We vitrified freshly purified, DDM-solubilised SctV on holey grids with an overlaid continuous film of amorphous carbon (Fig. S1D). 2D classification of the particles showed clear C9 symmetry in top view, consistent with our SEC profile, as well as the TMD and SctV_C_ domains clearly separate in side views (Fig. S1A, S1B). Reconstruction of the structure of SctV resolved structural details of the cytosolic ring, while we could not resolve the TMD of SctV_N_ despite signal subtraction and TMD-focused local alignment attempts. Our inability to align micelle features suggests the TMD is disordered or has high variability of conformation in the DDM micelle. In our hands, exchanging SctV from DDM to Amphipol A8-35 resulted in a more compact TMD density in preliminary cryoEM data (Fig. 1B, S2C), though the amphipol-stabilised SctV posed significant challenges to grid optimisation, with low yield and attraction to the air–water interface (Fig. S2A, S2B). The amphipol-stabilised SctV data is not directly comparable to the DDM data due to lower data quality and a lack of side view particles, however the more compact TMD signal better matches the *in situ* density presumed to be SctV_N_ (Butan et al. 2019), although TMD signal is still weaker than SctV_C_ signal in amphipol data (Fig. S2C). Our result is not sufficient to conclude whether SctV_N_ is ordered in A8-35 amphipol. In DDM, SctV_N_ does appear disordered with a minority of particles sharing some common micelle features and possible protein density extending above it, none of which could be used for alignment despite a variety of approaches (Fig. S2D).

2D classification of amphipol-stabilised SctV revealed partial arcs, possibly the result of particle disassembly at the air–water interface (Fig. S2B). These partial arcs retained the same radius of curvature of complete rings, indicating that self-assembly of the nonameric complex of cytoplasmic domains is dictated by inflexible intersubunit interactions, and closed rings were assembled exclusively from nine subunits.

By further processing the DDM-SctV dataset, focusing on the cytosolic domain and imposing C9 symmetry, we obtained a final map at 3.5 Å global resolution as judged by gold standard half-map FSC at a 0.143 threshold (Table S1, Fig. S3A). Local resolution ranged from 3.3 to 4.6 Å in the modelled regions of the map (Fig. S3B, S3C) and could not be assessed for the TMD due to the absolute lack of recognizable features in the reconstruction.

### 3.3 Structure of SctV_C_

Our 3-D reconstruction allowed us to build a model of SctV_C_ using a homology model and interactive modelling with ISOLDE (Croll, 2018) (Table S1, Fig. 1C). We were able to model all but the last nine residues (673–681) (Fig. 1E), revealing four subdomains, SD1 (residues 350–418 and 464–479), SD2 (residues 419–463), SD3 (residues 480–569), and SD4 (residues 570–672) (Fig. 1E) as predicted (Yu et al., 2018). This subdomain arrangement is conserved across the SctV/FlhA family (Saijo-Hamano et al., 2010, Worrall et al., 2010, Moore and Jia, 2010, Bange et al., 2010, Abrusci et al., 2013, Majewski et al., 2020).

SctV_C_ forms a homononameric torus, consistent with previously published structures of homologs (Abrusci et al., 2013, Butan et al., 2019, Kuhlen et al., 2020, Majewski et al., 2020). The protomers are held together by intermolecular SD3-SD3 (E502-R490, E506-N514, R534-E482, R534-E488, D542-R522, R546-E518, R563-E482) and SD3-SD1 (R531-Q406, R534-Y405, Y500-Y405, R563-E407) salt bridges and H-bonds, and hydrophobic contacts between the linker domain and the membrane facing area of SD1 in the neighbouring domain (P348-V347-A495, M346-W375-F378) (Fig. 1D) as previously observed in crystal structures (Saijo-Hamano et al., 2010, Bange et al., 2010).

SD3, which lines the inner surface of the ring, showed the highest resolution features, likely due to circumferential SD3-SD3 interactions with neighbouring protomers to form a continuous ring of nine SD3s with low flexibility; the resolution of the map decreased radially toward the outside of the ring (Fig. S3C). The resolution of the outer face, mainly composed of SD2 and SD4, was anisotropic perpendicular to the plane of the ring; 3-D classification of the final subset of particles did not separate distinct conformational differences, and this anisotropy is consistent with continuous motion of SctV between open and closed states represented in conformations seen in structures of other members of the family (Saijo-Hamano et al., 2010, Inoue et al., 2019).

The SctV residues reported to be important for gatekeeper function in the SPI-2 T3SS, F378, E488, R509, K515, R531, R590, E591, S592, I593, T596, V632, and D633 (Yu et al., 2018), map to the inner surface of the ring (Fig. 1F). Most are clustered in clefts on the cytoplasmic face of the ring between neighbouring SD4s, including V632, a residue whose mutation abolishes interaction of gatekeeper and SctV in both SPI-2 and SPI-1 T3SSs (Yu et al., 2018). These data suggest that the gatekeeper binds between the clefts formed by neighbouring SD4s to regulate the second secretion switch. This candidate position is distinct from the SD2-SD4 cleft binding of late flagellar substrate-chaperone complexes to FlhA, the flagellar homolog of SctV (Xing et al., 2018).

The C-terminal tail of SctV^SPI-2^ is an acidic sequence of 9 residues (673 – EEELADNEE – 681) that extends from SD4 and is unique among the SctV family (Fig. S3E). Its deletion partially mimics the phenotype of *SsaL* (SctW) mutants, suggesting that it is involved in the regulation of the second switch (Yu et al., 2018), and thus, possibly, in gatekeeper binding. Its position would be in close proximity to gatekeeper complexes if they bind at the bottom surface of the SctV_C_ ring between neighbouring SD4s. Although local resolution at the extremity of SD4 is poor (4.5 Å), we saw no density to build the C-terminal tail of 9 residues nor the 6 histidine tag beyond it. Secondary structure prediction software predict either ɑ-helical (PSIPRED 4.0 (Jones, 1999) and RaptorX (Wang et al., 2016)) or disordered structures (Jpred 4 (Drozdetskiy et al., 2015), PredictProtein (Yachdav et al., 2014)) (Fig. S3F). In light of the lack of density in the reconstructed map, these predictions support an interpretation that the acidic C-terminal tail of SctV^SPI-2^ is disordered, and further predict some propensity to form alpha helix.

### 3.4 Simulation shows SctV_C_ hinging open and closed

**Fig. 2:**
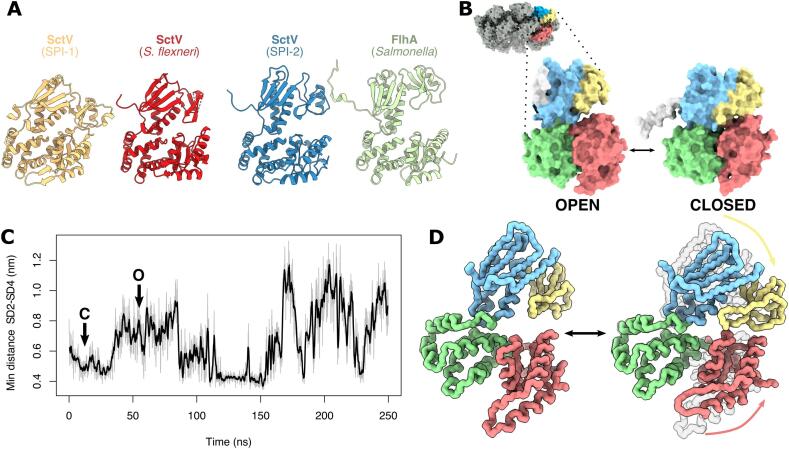
SctV_C_ hinge-like motion. A. Comparison of degree of hinge opening in different structures of SctV/FlhA family proteins. All of them are equally oriented after a local alignment of their respective SD3s. PDB accession codes: SctV^SPI-1^ (2X49), *Shigella flexneri* SctV (4A5P), *S. enterica* FlhA (3A5I). B. Central structures of the classes found by cluster analysis of the conformations adopted by SctV_C_ during simulation using a threshold of 4 Å.The structures are extracted from the 250 ns simulation at times t = 12 ns (right) and t = 50 ns (left). The monomer is placed in the context of the nonameric structure in the top-left inset. C. Minimum distance between subdomains 2 and 4 over simulation time in simulation 1 - pH 7.2. D. Graphic representation of the first motion component detected in the PCA. For simplicity only the backbone of the SctV_C_ is shown. The arrows indicate the direction of the motion. A movie of this motion is also available (Supp. movie 2). The subdomains in B and D are coloured as in Fig. 1.

Although the subdomain topology is conserved in all SctV/FlhA members, the relative orientations of SD2 and SD4 varies among the available structures (Saijo-Hamano et al., 2010, Worrall et al., 2010, Moore and Jia, 2010, Bange et al., 2010, Abrusci et al., 2013). This variation affects the accessibility of the inter-SD2-SD4 cleft, ranging from closed in a crystal structure (2X49) of SctV_C_^SPI-1^ to open in a crystal structure (3A5I) of *Salmonella*’s FlhA (Fig. 2A). Together with the smearing of SD2 and SD4 in our map, and SD2-SD4 motion in molecular dynamics (MD) simulations of FlhA (Saijo-Hamano et al., 2010, Inoue et al., 2019), these data suggest that hinging of SD2 and SD4 about the SD3 backbone is a common feature of all family members, modulating the accessibility of the SD2-SD4 cleft.

We used MD simulations to probe the dynamic nature of the cleft and any other conformational changes that may occur in SctV_C_^SPI-2^. A 250 ns simulation of our SctV_C_ monomer confirmed a hinge-like motion in which the SD2-SD4 cleft opened and closed (Video 1). We also detected this motion in four independent 100 ns simulations (Table S2). Clustering analysis of SctV_C_’s conformations revealed two main conformations, open and closed (Fig. 2B). We tracked the minimum distance between SD2 and SD4 over time, corroborating that the protein opens and closes in all replicates (Fig. 2C, Fig. S4A). For instance, at t = ~80 ns in our 250 ns simulation, SD2 and SD4 subdomains closed, reopening at t = ~150 ns (Fig. 2C).

The narrowing of the SD2-SD4 cleft during the simulation is the result of hinging about SD3. SD3 remained essentially rigid in all simulation replicates, independent of the position of SD2 and SD4 as shown by the root-mean-square fluctuation (RMSF) (Fig. S4B). The hinging motion is the main conformational change experienced by SctV_C_ in the simulations and principal component analysis (PCA) of SctV_C_ dynamics revealed the opening and closing of the SD2-SD4 cleft as the first principal component (Fig. 2D, Suppl. Video 2). That monomeric SD3 remains rigid, together with its role as a stable hinge, and its many interactions with neighbouring protomers in the nonameric ring explains its high sequence conservation and it being the best resolved part of the structure (Fig. S4C).

### 3.5 Consequences of acidification

**Fig. 3:**
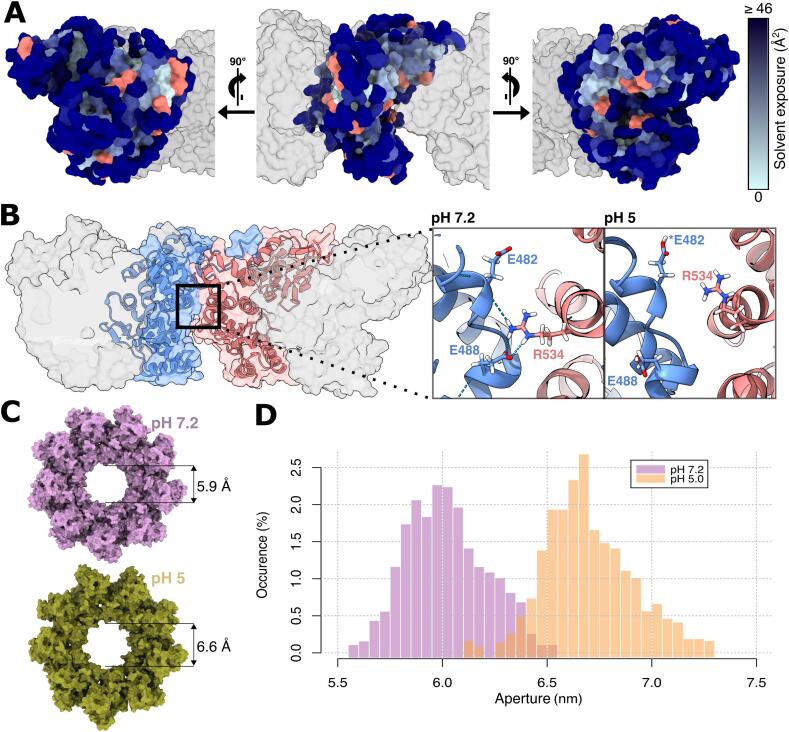
Effect of acidification on SctV_C9_. A. SctV_C_ monomer surface rendering coloured by residue area of solvent exposure (light to dark blue). The predicted protonation sites at pH 5.0 are highlighted in red. B. Overview of the interface between protomers of the ring. In the inset, detailed visualisation of the salt-bridge network between E482, E488 and R534. The asterisk indicates the protonation of E482. C. Representative snapshots of SctV_C9_ during MD simulations at pH 7.2 and pH 5.0 with median ring apertures. D. Distribution of ring apertures during SctV_C9_ MD simulations, measured as the distance between R497 and L499 in chains opposed in the ring, such as A and E.

Given that the cytoplasmic pH of *Salmonella* acidifies while residing in the acidified SCV lumen (Chakraborty et al., 2015, Liew et al., 2019) and that the second secretion specificity switch in the SPI-2 T3SS is regulated by a shift from acidic to neutral pH (Yu et al., 2010, Yu et al., 2018), we investigated how SctV_C_ dynamics differ between acidic and neutral pH. We determined the protonation state of our nonameric SctV_C_ model at pH 5.0 and pH 7.2 by predicting which residues would be protonated in each condition and optimising the H-bond network around them using ProteinPrepare (Martínez-Rosell et al., 2017) for SD1 (H362 and E407), SD2 (E452), SD3 (E482, E502, E506, E518, D532, D542, E547, and H564) and SD4 (E586, H608, E643, E656, and E667). Some of the residues predicted to be differentially protonated between states coincides with those involved in interprotomeric stabilisation (E407, E482, E502, E506, E518, and D542), indicating that the cytoplasmic pH shift may affect nonamer stability. By mapping these residues to our SctV_C_ structure, it is evident that their positions enclose areas buried in the interprotomer interface (Fig. 3A). We ran 80 ns MD simulations of nonameric SctV_C_ in both protonation states, with two shorter replicates for each state (Table S2). We observed higher mobility at acidic pH than at neutral pH, as expected due to loss of interprotomer interactions by protonation of interacting residues (Fig. S4E). A representative example of the loss of interactions at low pH is the H-bond network present at pH 7.2 between E482, E488 and R534, that was maintained during the whole simulation in at least eight out of nine interprotomeric interfaces in all four replicates at pH 7.2. At pH 5, however, protonation of E482 disrupted these interactions in all replicates (Fig. 3B). These data suggest that the interfaces between protomers would be substantially affected if *Salmonella*’s cytoplasm or the local environment of SctV_C_ becomes more neutral upon assembly of the translocon in the SCV membrane.

The comparison between simulations of nonameric SctV_C_ in acidic and neutral pH conditions revealed that the ring is more flexible at low pH. The number of SD3-SD3 interactions decreased due to the protonation of some residues involved in electrostatic interactions, resulting in a more mobile SD3 (Fig. S4F). This reduced rigidity resulted in an increase of the nonameric ring’s aperture due to expansion of the subunits in the ring plane together with a perpendicular “buckling” motion (Fig. 3C). The distribution of the aperture sizes during simulation (Fig. 3D, Fig. S5) illustrates how the loss of SD3-SD3 interactions at acidic pH resulted in increased flexibility, although sufficient interactions were retained to maintain the nonameric assembly.

We also simulated monomeric SctV_C_ at pH 5 to investigate whether differences in protonation affected SD2-SD4 hinging (Table S2). We observed higher RMSD early in the simulation due to equilibration of a model derived from the neutral pH structure subsequently simulated at pH 5 Fig. S4E). We did not, however, detect differences in SD2-SD4 hinging motion compared to the simulations at pH 7.2 (Fig. S4D).

## 5 Discussion

SctV is the injectisome component that dictates substrate specificity. It binds secretion substrates and, together with the export apparatus components SctRSTU, translocates substrates across the inner membrane and into the hollow needle that traverses periplasm, outer membrane and, via a translocon pore, the host cell membrane (Fig. 1A). The mechanism that, in coordination with the gatekeeper, results in a change of specificity from translocon to effector substrates is still far from clear. Aiming to better understand this mechanism, here we present the cryo-EM structure of SctV_C_^SPI-2^ from purified full-length protein.

While we were able to model the structure of SctV_C_, we were not able to determine the structure of SctV_N_. How SctV_N_ interacts with the rest of the secretion machinery remains a gap in our understanding of T3SSs, and more work is needed to assess whether the interaction with the air–water interface and orientation bias problems experienced with the amphipol-SctV sample can be overcome, and whether reconstitution with amphipol permits stabilization or refolding of an apparently disordered TMD in detergent micelles. Given SctV_N_ assembles with SctRSTU forming a secretion lumen in close proximity (Kuhlen et al., 2020), it is possible that sufficient stabilisation of the TMD is dependent on its interaction partners, requiring difficult reconstitution of many components or *in situ* approaches (Butan et al., 2019).

SctV_C_^SPI-2^ oligomerizes as a homononameric ring in solution. The complex is held together mainly by electrostatic interactions between neighbouring SD3s and between the linker domain and a hydrophobic pocket in SD1. This results in a ring of SD3 domains forming a stable backbone around the inner face of the ring, about which SD2 and SD4 hinge. As we observed in our simulations, this motion allows SctV to open and close, resulting in substantial variation in accessibility to the SD2-SD4 cleft where late flagellar secretion substrates have been reported to bind (Xing et al., 2018). In our simulations the cleft appears bistable between open and closed, raising the possibility of one state or another being stabilised, although neither pH 5 nor 7.2 alone stabilised either state, though interacting chaperones or gatekeeper might.

Previous reports indicate that the gatekeeper binds SctV upon exposure to acidic conditions (Yu et al., 2010, Yu et al., 2018) and acidification of *Salmonella*’s cytoplasm in the SCV, and then dissociates upon exposure to neutral pH (Yu et al., 2010). Taken together, this might suggest that residues directly involved in SctV-gatekeeper docking are differentially protonated under different pH conditions. Our protonation predictions, however, suggest that most differentially-protonated residues map to the interprotomeric interfaces of the ring, rather than to its inner or outer face. This suggests a different model: the different protonation states recruit the gatekeeper by inducing conformational changes in the SctV_C_ ring. In support of this mechanism, the comparison of our simulations in both conditions shows how, as a result of the loss of some of the interactions between monomers due to protonation at pH 5, interprotomeric interactions are disrupted, resulting in a more flexible ring. On the other hand, most of the SctV residues reported to be involved in gatekeeper function (and thus possibly binding) are clustered in the inner face of SD4 and along the clefts on the bottom of the ring formed by the interfaces between monomers. This includes V632, a single residue whose mutation reproduces the gatekeeper knockout phenotype in both SPI-2 and SPI-1. Both observations are compatible with gatekeeper being recruited to the interface area upon acidification in response to the relaxation of the interactions that keep together neighbouring monomers. Nevertheless, we cannot discard the possibility of some differentially protonated residues, both in gatekeeper and SctV being implicated in this interaction. Although the injectisome T3SS machinery is well conserved across evolution, the mechanism of regulation of the second specificity switch might not. The interaction between gatekeeper and SctV in enteropathogenic *E. coli* (EPEC) seems to occur in the outer face of the ring (Portaliou et al., 2017), in contrast with our conclusions. The actual mechanism behind the gatekeeper’s regulation of the secretion is either not conserved between EPEC and SPI-2 or we are describing different facets of a more complex phenomenon. Indeed, we cannot rule out the possibility that pH sensing is extrinsic to SctV, and may instead be sensed distally and transmitted to SctV such as via an allosteric mechanism through the needle (Guo et al., 2019) and SctDJ (Butan et al., 2019, Umrekar et al., 2020). Alternatively, unless gated, the translocon pore being large enough to export unfolded proteins could easily permit a change in pH to transmit within the secretion channel and this might extend to the local area of SctV at the base of the secretion channel.

Our work also provides other findings. The unique SctV_C_^SPI-2^ C-terminal extension, important for gatekeeper function, is either disordered or dynamic, as demonstrated by the missing density of the map in this area and our secondary structure predictions. We can also explain the higher sequence conservation of SD3 compared to the rest of the cytoplasmic domain, given that it is key in driving SctV_C_ oligomerisation and acts as a stationary hinging point for SD2 and SD4. Finally, our Amphipol-SctV dataset, that features a considerable number of broken particles due to destructive interactions with the air–water interface, preserves its radius in broken arcs and indicates that the assembly is intrinsically nonameric and that the interactions between nonamers are strong and determine a precise interface angle. This observation supports self-assembly of FlhA without an extrinsic scaffold, unlike SctDJ, whose oligomeric state depends upon the presence of SctRST (Butan et al., 2019).

**Fig. 4:**
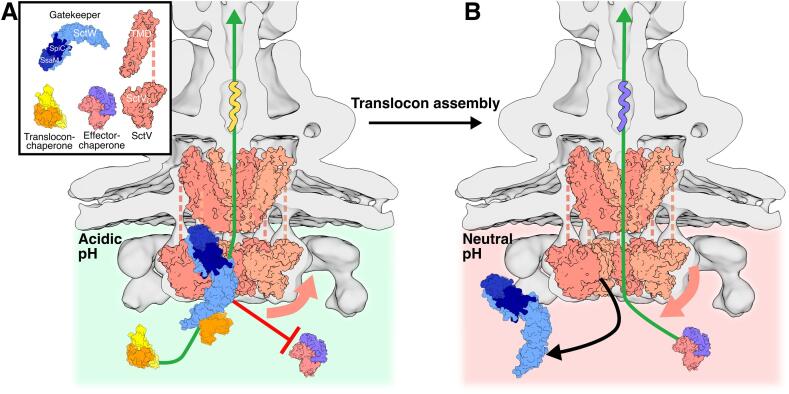
A model for the mechanism of regulation of the second secretion switch. A. The acidic pH of *Salmonella*’s cytoplasm results in the protonation of several residues involved in SctV interprotomeric interactions, preventing the tight association between neighbouring subunits. This provides enough flexibility in the cytoplasmic domain of SctV_9_ (pink arrow) for the gatekeeper complex to bind it. Gatekeeper likely recognises chaperone-substrate complexes via the chaperone moiety (Archuleta et al., 2014), facilitating the secretion of translocon subunits across the needle (green arrow). It also prevents the secretion of the pathogenic effectors (red arrow). B. The assembly of the translocon subunits forming a pore in the SCV membrane establishes a connection between the cytoplasm of host and *Salmonella* across the injectisome. This connection may allow a local increase in pH around the export gate, causing deprotonation of residues at the interprotomeric interfaces of SctV_9_ and formation of salt bridges that tighten and stabilise the ring (pink arrow). This conformational change releases or blocks gatekeeper complex (black arrow), thus enabling the injectisome to translocate pathogenic effectors into the host cytoplasm (green arrow). Injectisome map: EMDB 20838. Gatekeeper model: composite model of 1XL3 and 1XKP. Translocon subunit: 3TUL. Chaperone: 4NRH. Effector: 5HAF. SctV TMD: from (Taylor et al., 2016).

Our findings are compatible with SctV_C_ conformation being affected by the pH change that triggers the translocation of pathogenic effectors to the host cytosol. After host cell uptake, *Salmonella* is confined to the SCV. The decrease of the pH of the SCV lumen (Drecktrah et al., 2006) leads to the acidification of *Salmonella*’s cytoplasm (Chakraborty et al., 2015), triggering the expression (Liew et al., 2019) and assembly of the components of the SPI-2 injectisome (Beuzon et al., 1999). Under acidic pH conditions of the SCV, interaction between SctV_C_ protomers in the nonameric ring is sufficiently weak to provide individual subunits freedom of motion that remodels the interprotomeric regions to be more exposed. The cleft between monomers at the inner face of the ring is likely to be the gatekeeper binding region (Yu et al., 2018). Thus, this conformational change may expose the necessary residues for this interaction. Gatekeeper binding to SctV_C_ facilitates export of translocon subunits and blocks effector translocation (Yu et al., 2018). Once the translocon subunits are assembled into the SCV membrane, a connection between the host cell and *Salmonella’*s cytoplasm is established through the needle, and gatekeeper dissociates from SctV_C_ (Yu et al., 2010, Yu et al., 2018). SctV_C_ may directly sense this pH change, change conformation, and tighten its SD3 interprotomeric interactions, denying gatekeeper access and allowing translocation of virulence proteins (Fig. 4).

## 6 Conclusions

Our study is one of a new wave to apply cryo-EM to determine the structure of a SctV family member in its nonameric state under native-like conditions (Majewski et al., 2020). In this case, SctV was extracted from membranes as a full-length membrane bound complex resembling its condition *in situ*, although only its cytoplasmic domain could be resolved while the TMD appears disordered when solubilised in DDM. We also used MD to explore the dynamic properties of our model, observing a hinge-like motion concerning SD2 and SD4, as well as pH-dependent remodeling of the interchain interactions in the nonamer.

Our structural and dynamic studies lead us to propose a mechanism of regulation of the SPI-2 translocon to effector specificity switch in which pH sensing involves SctV_C_. Since the mechanisms of specificity switching appear diversified among different T3SSs (Gaytán et al., 2018), in order to understand the role of the gatekeeper in these other systems, it will be crucial to determine rigorously where and how gatekeepers bind these SctV assemblies.

## Author contributions

TRSM-P and MB conceived and designed the research. DH, GG, and X-JY generated the SctV expression strain. SL, TC, TRSM-P and TZ purified SctV. AN, PBR and TRSM-P collected cryo-EM data. TRSM-P processed the cryo-EM data. NG-R and TRSM-P built the SctV_C_ atomic model. NG-R and SLR performed MD simulations and analysed the MD data. MB, NG-R, SL and TRSM-P analysed the data. NG-R, TRSM-P, and MB created the figures and wrote the manuscript. All authors reviewed and agreed on the content of this article.

## Funding sources

TRSM-P was supported by the 10.13039/100010438Francis Crick Institute (which receives its core funding from 10.13039/501100000289Cancer Research UK [FC001179], the UK Medical Research Council [FC001179], and the 10.13039/100010269Wellcome Trust [FC001179]), SLR was supported by Medical Research Council Grant MR/T017961/1, and MB was supported by Medical Research Council grant MR/P019374/1.

## Declaration of Competing Interest

The authors declare that they have no known competing financial interests or personal relationships that could have appeared to influence the work reported in this paper.
