# Supplementary material for: Structure of the cytoplasmic domain of SctV (SsaV) from the Salmonella SPI-2 injectisome and implications for a pH sensing mechanism
Source: J Struct Biol. 2021 Jun;213(2):107729. doi: 10.1016/j.jsb.2021.107729 (PMC8223533; doi:10.1016/j.jsb.2021.107729)
Supplement: Supplementary data 1 [file mmc1.docx]

# Supplementary materials

**
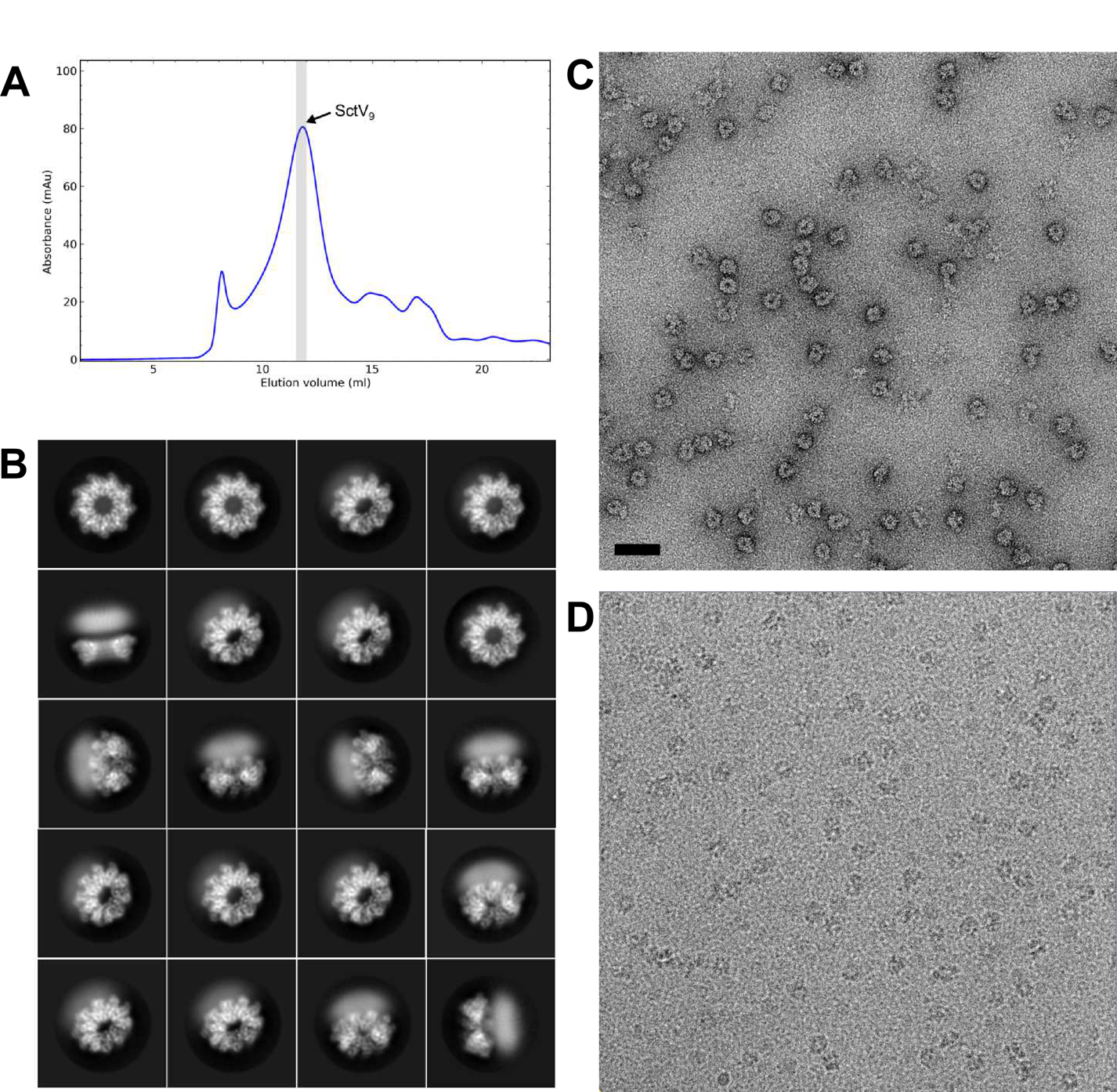
**

**Figure S1: SctV_9_ purification and cryo-EM. A.** Size-exclusion chromatography profile of DDM-SctV_9_. The fraction coloured in grey was used for negative stain EM imaging**. B.** 2D classification averages of DDM-SctV^SPI-2^ particles showing top, tilted and side views. **C.** Representative negative stain EM micrograph of eluted DDM-SctV_9_ rings. Scale bar: 50 nm. **D.** Representative micrograph of vitrified DDM-SctV^SPI-2^ dispersed on a thin layer of amorphous carbon. **E.** 2D classes of A8-35-SctV^SPI-2^ showing more compact TMD and partial arcs.


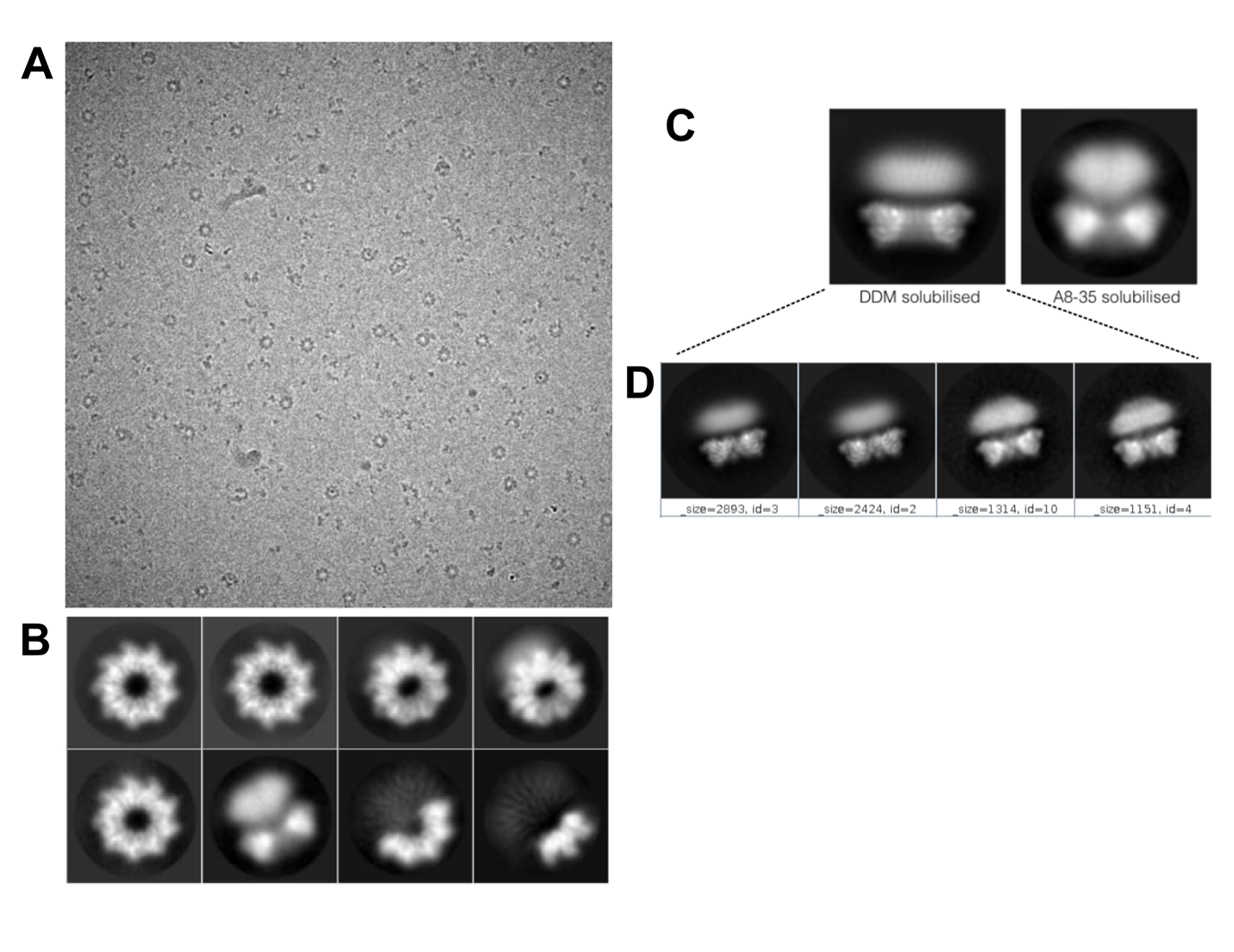
**Figure S2: Amphipol stabilisation and TMD comparison. A.** Representative micrograph of A8-35-SctV^SPI-2^ particles in ice in holes. **B.** 2D classification averages of A8-35-SctV^SPI-2^ particles showing more compact TMD and partial arcs of broken particles. **C.** Comparison of side views of DDM and A8-35 solubilised SctV^SPI-2^. **D.** Classification, without searching alignment, of DDM-SctV^SPI-2^ side views from the class in C. shows that the TMD appears as varying shapes. Faint micelle features exist in a subset of the data, but no features were recovered with local alignment and signal subtraction approaches.

| **Data collection** | |  | **Atomic model** | | |
| --- | --- | --- | --- | --- | --- |
| Electron microscope | Titan Krios |  | Ramachandran plot (%) | | |
| Electron detector | Gatan K2 |  |  | Outliers | 0.00 |
| Voltage (kV) | 300 |  |  | Allowed | 5.83 |
| Pixel size (Å) | 1.08 |  |  | Favoured | 94.17 |
| Defocus range (μm) | 1.5 - 4 |  | Clash score | | 6.58 |
| Fluence (e-/Å^2^) | 70 |  | Molprobity score | | 1.76 |
| Number of raw movies | 6729 |  | Rotamer outliers (%) | | 0.00 |
| **3-D reconstruction** | |  | Cβ outliers (%) | | 0.00 |
| Symmetry | C9 |  | Bonds RMS deviations | | |
| Initial number of particles | 988000 |  |  | Lengths (Å) (#>4σ) | 0.004 (0) |
| Final number of particles | 331840 |  |  | Angles (º) (#>4σ) | 1.008 (0) |
| Resolution FSC = 0.143 (Å) | 3.5 |  | **Model-to-map agreement** | | |
| Resolution range (Å) | 3.3 - 4.8 |  | Resolution FSC = 0.5 | | 3.7 |
| Map sharpening B-factor (Å^2^) | -100 |  | CC | | 0.81 |

**Table S1**: **Cryo-EM, image processing and model building parameters and statistics.**

**
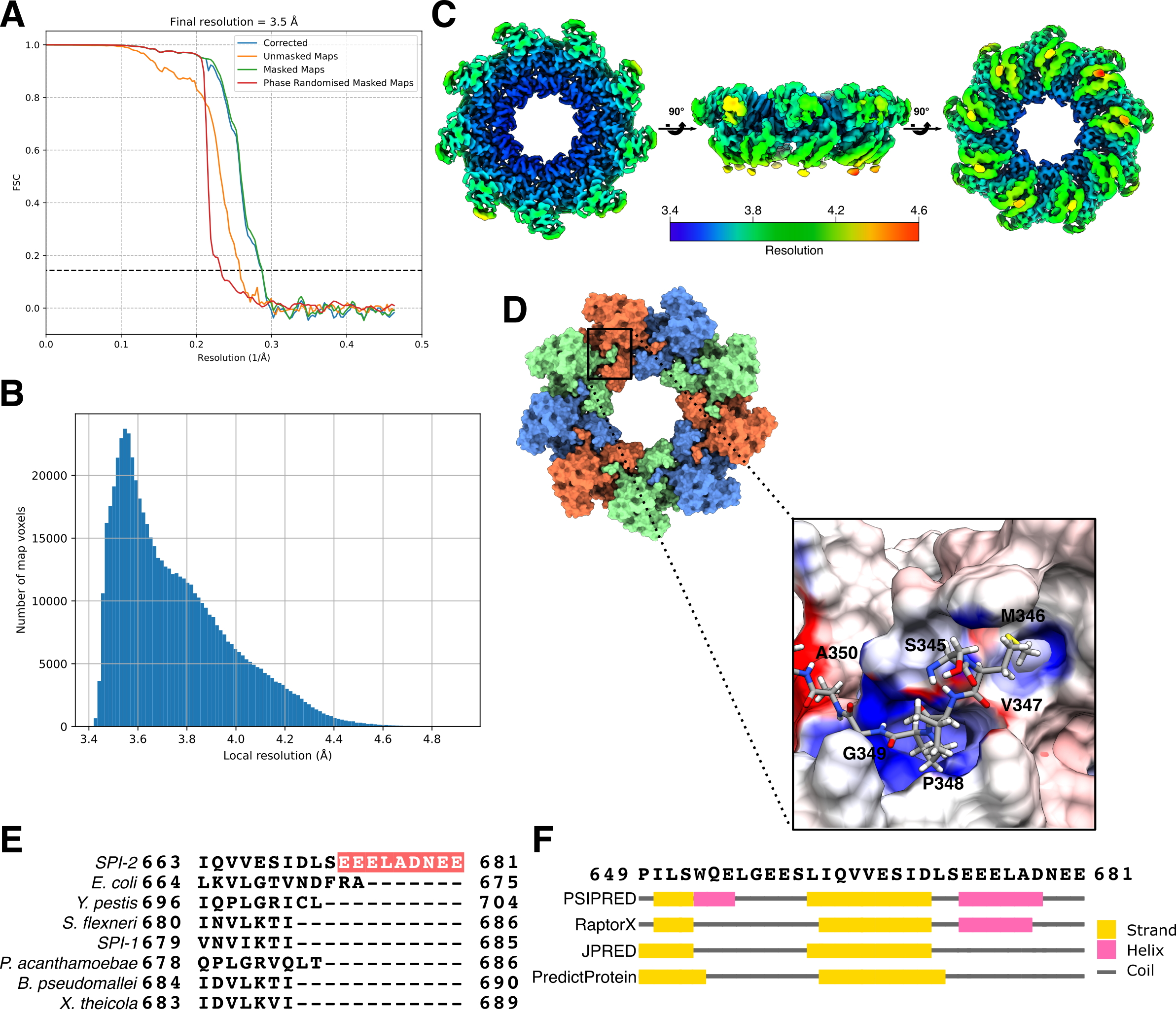
**

**Figure S3: SctV_C_ resolution and structural details. A.** FSC between two independently refined half-maps. **B.** Local resolution histogram. **C.** Local resolution map. **D.** Detail of the interaction between linker domain (stick rendering) and hydrophobic pocket (surface rendering) in the membrane-facing region of SD1. Hydrophobicity is rendered from blue (hydrophobic) to red (hydrophilic). **E.** Multiple sequence alignment of the C termini of SctV family members. SctV^SPI-2^ has a unique, acidic C-terminal tail. **F.** Secondary structure predictions of the SctV^SPI-2^ C-terminal tail.

| **Atomic model** | SctV_C_ (monomer) | | | | | | | | SctV_C9_ | | | | | |
| --- | --- | --- | --- | --- | --- | --- | --- | --- | --- | --- | --- | --- | --- | --- |
| **pH** | 5 | | | | 7.2 | | | | 5 | | | 7.2 | | |
| **Replicate (#)** | 1 | 2 | 3 | 4 | 1 | 2 | 3 | 4 | 1 | 2 | 3 | 1 | 2 | 3 |
| **Length (ns)** | 250 | 100 | | | 250 | 100 | | | 66 | 80 | 72 | 80 | 80 | 72 |
| **Box size (nm x nm x nm)** | 20 x 20 x 16 | | | | | | | | 12 x 12 x 12 | | | | | |

**Table S2**: **Molecular dynamics simulations summary**.

**
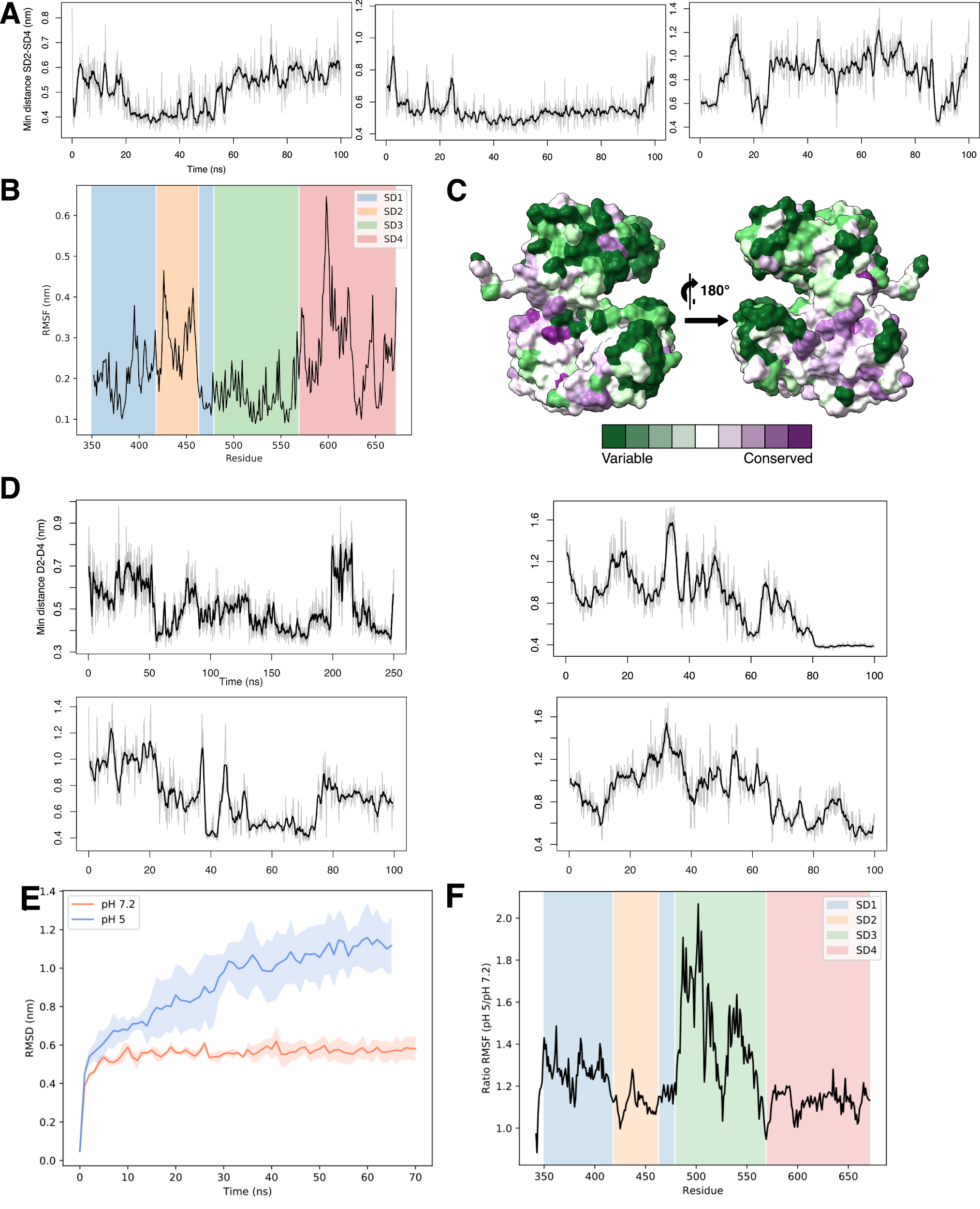
**

**Figure S4: SctV_C_ dynamics and conservation. A.** Minimum distance between subdomains 2 and 4 over simulation time in pH 7.2 simulation replicates 2 (left), 3 (center) and 4 (right). **B.** Root-mean-square fluctuation (RMSF) of SctV_C_ during simulation 1 - pH 7.2. **C.** Conservation map of SctV_C_, showing SD3 as the best conserved part of the protein. **D.** Minimum distance between subdomains 2 and 4 over simulation time in pH 5.0 simulations 1 (top left), 2 (top right), 3 (bottom left) and 4 (bottom right). **E.** Comparison of the RMSD change over time in the SctV_C9_ MD simulations at pH 7.2 (red) and pH 5.0 (blue). Solid lines indicate the average of the three replicates, the pale shadow indicates the standard deviation. **F.** pH 5/pH 7.2 ratio of the root-mean square-fluctuation of each residue. Although it is the less mobile region of the protein in both conditions, SD3 mobility increases at pH 5. The data was calculated from replicates 2 for being the longest ones (Table S2).


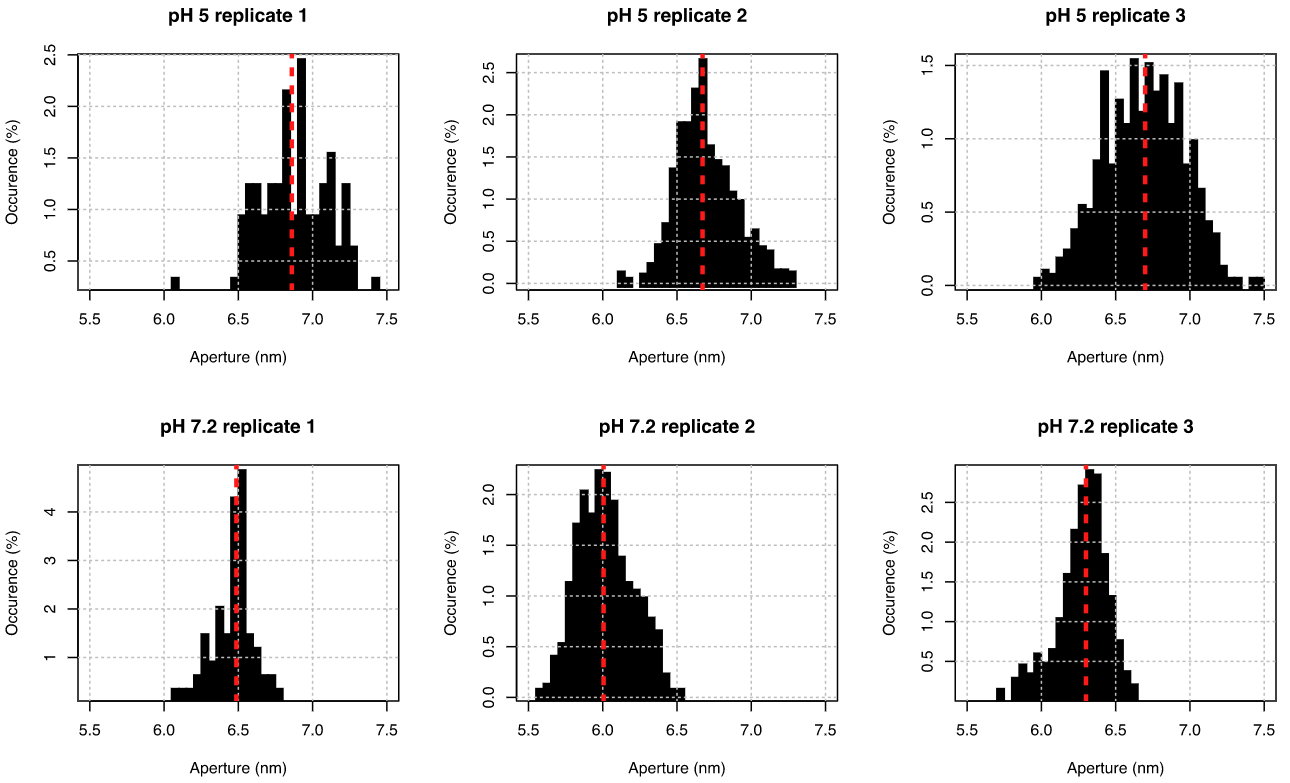


**Figure S5: Aperture distributions in all nonameric SctV_C_ replicates.** Red dashed lines indicate the median aperture.
